# Supplementary material for: Deep template-based protein structure prediction
Source: PLoS Comput Biol. 2021 May 3;17(5):e1008954. doi: 10.1371/journal.pcbi.1008954 (PMC8118551; doi:10.1371/journal.pcbi.1008954)
Supplement: Attachment — Submitted filename: NDThreader-ReplyLetter.docx [file pcbi.1008954.s005.docx]

Dear PLoS Computational Biology Editor,

We thank you and the reviewers very much for thorough and insightful reviews of our manuscript and for the opportunity to revise it. According to the reviews, we have improved our manuscript (with revisions marked in red) and made our software publicly available for non-commercial usage. Below please see our point-to-point reply to the reviews which are displayed in *Italic*.

*Editor comments:*

*Both reviewers clearly appreciate the reported performance and the clarity of the manuscript. However, the second reviewer raises an important concern about the question of reproducibility and method availability. Currently only a link towards the RaptorX server is provided, but all seems to be integrated and not independently testable. Please address this point carefully.*

Answer: our protein threading programs (DRNF and NDThreader) can be downloaded at <http://raptorx.uchicago.edu/Download/> . After login, open file 0README.DL4Threading.txt and follow the instructions for download and installation. The code for building 3D models from an alignment is available in our template-free modeling package [RaptorX-3DModeling](https://github.com/j3xugit/RaptorX-3DModeling) . Our plan in the near future is to integrate the threading modules into RaptorX-3DModeling. The list of protein pairs for training/validation/test has been provided in Supplemental.

*Reviewer's Responses to Questions*

***Comments to the Authors:***

***Please note here if the review is uploaded as an attachment.***

*Reviewer #1: The authors improve template-based modeling over their previous method by using ADMM to align predicted distance potential to that of a template. Overall good description of method and what was done during CASP.*

*Some general concerns:*

*1) The authors state that "DeepThreader is the first distance-based threading". But it seems DALI (Distance-matrix ALIgnment method) could also be used to align distance matrices. Have authors tried using predicted distance matrices with DALI for template search/ranking and threading?*

Answer: DALI is a protein structure alignment program based upon the alignment of two distance matrices while DeepThreader is a protein threading method. DeepThreader and DALI are common in using distance matrices, but different in that DeepThreader uses the predicted distance matrix for a query sequence while DALI uses native distance matrices for both proteins. Each entry in the predicted distance matrix is a discrete probability distribution of distance. DALI cannot take such a distance probability distribution as input, so technically it is very hard to test if DALI may work with our predicted distance matrix or not. Further, DeepThreader also uses some 1-dimensional information such as primary sequence, sequence profile, predicted secondary structure and solvent accessibility (which are useful for TBM targets), but DALI does not.

*2) For cases where DeepThreader did better than NDThreader, is this because NDThreader got stuck in local minimum during ADMM alignment? If the best possible alignment (using TMalign) or alignment from DeepThreader is rescored using NDThreader's score function, does it have a better or worse score than the NDThreader's solution?*

Answer: it is possible that NDThreader may get stuck in a local minimum for some protein pairs and thus, lead to worse performance than DeepThreader. However, there is no guarantee that the NDThreader scoring function may score well on the alignments produced by TMalign and DeepThreader since they generate alignments by optimizing very different scoring functions.

*3) For Table 5, it would be good to see a scatterplot comparing the methods. Averages can be a bit misleading, as a single outlier target can skew the results. It would be good to know what fraction of the targets RaptorX did better than Zhang-Server/BAKER-SERVER. The statement in the abstract "the best GDT score among allCASP14 servers on the 58 TBM targets." makes it sound like the method got the best GDT score on all the 58 targets... though I'm guessing this only true on average?*

Answer: Sorry for confusion. We indeed meant the average performance on the 58 TBM targets. We added the below figures in the main text to show the head-to-head comparison of RaptorX vs. BAKER-RosettaServer and RaptorX vs. Zhang-Server on CASP14 TBM targets. In terms of TMscore, RaptorX outperforms Zhang-Server and RosettaServer on 28 and 24 targets (out of 58), respectively, and underperforms on 22 and 27 targets, respectively. In terms of GDT score, RaptorX outperforms Zhang-Server and RosettaServer on 36 and 28 targets, respectively, while underperforming on 22 and 30 targets, respectively. Compared to the other two servers, RaptorX generated much better 3D models on two targets T1030-D1 and T1099-D1 than the other two servers.

*Reviewer #2: The authors of "Deep Template-based Protein Structure Prediction" present a highly successful deep learning method for improving the accuracy of template based modelling of protein structures.*

*The paper is written in a readable, methodical way and is sufficiently easy to follow for an interested reader. The methods presented are both interesting and sufficiently novel.*

Answer: thanks a lot for being interested in our work.

*They would be of great use to the general community, if only it were available. In a current format, the article describes the method, though in a way that is not sufficient to replicate it by the reader.*

*= Major concerns:*

*1. The paper introduces new architecture - Deep Convolutional Residual Neural Fields (DRNF), which appears exciting and (judging by authors' report) performs exquisitely well for the problem approached. However, there is no reference implementation of this architecture available, not to speak about the actual method that could be used on arbitrary data. Without ability to replicate results, the message of the paper becomes much weaker.*

*2. Authors provide the compositions of the training/validation/testing set, but these are all the data provided. As there is no method available, there is no way to verify the correctness of the results, nor there is a way to test the method independently. Furthermore, there is no way to assess the quality of predictions beyond the metrics provided by the authors.*

Answer: See our answer to the editor for the availability of the software and data. The correctness of our method has been verified in CASP14.

*3. Authors of AlphaFold2 claim that their method largely supersedes TBM methods for protein structure prediction. How does DRNF and NDThreader compare to such a method - especially in terms of practical usefulness for the protein structure prediction and modelling communities?*

Answer: In CASP14, AlphaFold2 has also used templates (detected by HHpred) for some TBM targets although it is unclear for which targets AlphaFold2 has done so. My group in CASP13 started to integrate both template and coevolution information into a deep network for protein structure modeling. See section 2.6 in <https://onlinelibrary.wiley.com/doi/full/10.1002/prot.25810> for our method description. In CASP14, quite a few groups such as AlphaFold2 and Baker group have adopted such a strategy, i.e., feeding both template and MSA/coevolution information into deep networks. Our experimental results show that when the query is very similar to the template (>40% sequence identity), RosettaCM and MODELLER perform well. When the query is not similar to any templates (HHpred E-value > 1E-5), it is better not to use any template information. When the query and the template have sequence identity between 20% and 40%, on average template information complements to MSA/coevolution information especially when the MSA of the query sequence is shallow. Nevertheless, as expressed in our manuscript, for some TBM targets, our template-free modeling method may produce 3D models better than what can be built from both templates and coevolution information.

*4. Training/validation/test set separation. In p. 16 l. 273. "A multi-domain protein chain may belong to multiple groups". It is not evident for me, that such a partition does not result in an information bleed between data sets, especially in context of page 17 l. 285-288. Let's assume one protein pair contains domains in superfamilies S1 and S2, and is assigned to (let's say) training set by virtue of domain S1. Let's assume also that superfamily S2 is not a part of the training set (which, in light of the paper it does not need to be). Can the same protein pair be assigned to the validation/test set by virtue of domain S2? Can proteins containing domains in S2 be a part of validation/test sets, even though examples of these domains are already in training set?*

Answer: As described in section “Training and test data”, while generating the training, validation and test sets, we make sure there are no similar protein pairs between the test set and the training/validation sets. Two protein pairs are treated as similar if their query proteins share at least one superfamily IDs and so do their template proteins. This guarantees that a pair of multi-domain proteins will not appear in both the training and test sets.

*5. ADMM diversification, cf. p. 22. l. 411-413. How much of divergence can one expect with different initialization criteria? It would seem, like for the "easier" tasks inclusion of SAS and/or SS priors should be irrelevant. And both TMalign and DeepAlign should result in identical alignments... Can you provide an estimate how often do such a treatment help?*

Answer: The above figures show the impact of SAS/SS and the DeepAlign/TMalign alignments on DRNF’s performance. Indeed, for the ‘easier’ tasks all DRNF models generate very similar alignments. For those difficult pairs, SAS and SS help to improve the alignment accuracy. These two pictures have been added to Supplemental section 7, which contains the ablation study of DRNF under different settings. For those difficult pairs, multiple initial alignments for NDThreader may improve alignment quality. Our experimental results show that with 4 different initial alignments on average we may improve the alignment quality of NDThreader by 0.01~0.02 TMscore over a single initial alignment.

*= Minor concerns:*

*p. 1 l. 12/13 NDThreader becomes DNThreader*

Answer: thanks. We have fixed it.

*p. 1. l. 17 unnecessary space in 'co-evolution'*

Answer: we have fixed it.

*p. 2. l 30 "methods developed for TBM": missing verb*

Answer: we have fixed it.

*p. 2. l. 40 "good percentage" - would you care to specify?*

Answer: 67 out of 112 test domains in CASP13 and 58 out of 107 test domains in CASP14 have reasonable templates in PDB. In real-world applications, a larger portion of proteins shall have templates in PDB since CASP does not use the proteins with very good templates as test targets.

*p. 15. Table 3. for NDThreader at (TM+GDT)/2 "top 1" score value 0.419 is incongruent with the rest of the table. I assume it is a typo... Additionally, please look into the table headers and their alignment.*

Answer: thanks a lot for detecting this. We have fixed it.

*p. 22. l. 410. "converges to local optimal" - I think you mean "optimum"*

Answer: yep. We have fixed it.

*p. 22. l. 411-413. How much of divergence can one expect with different initialization criteria? It would seem, like for the "easier" tasks inclusion of SAS and/or SS priors should be irrelevant. And both TMalign and DeepAlign should result in identical alignments...*

Answer: we have answered this question above.
