# Supplementary material for: Deep template-based protein structure prediction
Source: PLoS Comput Biol. 2021 May 3;17(5):e1008954. doi: 10.1371/journal.pcbi.1008954 (PMC8118551; doi:10.1371/journal.pcbi.1008954)
Supplement: Attachment — Submitted filename: NDThreader-ReplyLetter-v2.docx [file pcbi.1008954.s006.docx]

Dear PLoS Computational Biology Editor,

We thank you and the reviewers very much for thorough reviews of our manuscript and for the opportunity to revise it. According to the reviews, we have improved our manuscript (with revisions marked in red) and made our software more easily accessible. Below please see our point-to-point reply to the reviews displayed in *Italic*.

*Editor comments:*

*Besides a number of very minor comments, Reviewer 2 raises major concerns with respect to the sharing of your code, which does not comply with open source standards. Please change access to the software such that it can be downloaded and used without registration.*

Answer: our software can be downloaded at <https://github.com/wufandi/DL4SequenceAlignment> without registration. This manuscript is being reviewed as a PLoS CB Methods paper instead of a Software paper. To the best of our understanding of the PLoS CB policy, although the software described in a Software paper needs to fully comply with open source standards, the software underlying a Methods paper is not subject to this (see <https://collections.plos.org/collection/compbiol-methods/>) although the authors are required to make the software publicly available. We have released our source code for public and non-profit usage and do not put any restriction on an academic or non-profit user. But we do think it is very reasonable to forbid a commercial user from using our software without explicit permission. Nevertheless, if PLoS CB requires that all software fully comply with open source standards regardless of the article type, we will be happy to fulfill this.

*Reviewer's Responses to Questions*

***Comments to the Authors:***

***Please note here if the review is uploaded as an attachment.***

*Reviewer #1: The authors have addressed most of my concerns. I would recommend acceptance.*

*DALI should be cited, in the context of "first distance-based threading method", regardless if one uses real vs. predicted distances.*

Answer: We have cited the DALI.

*Reviewer #2: Thank you for comprehensively addressing my questions. I sustain my opinion, that the work presented in this paper is of high quality, both in terms of novelty and implementation and constitutes a valuable addition to the knowledge in the field.*

Answer: Thanks a lot for being interested in our work.

*However, even though the Authors do provide the software presented in the paper, it does not fulfil the Open Source requirement of the Journal. It is impossible to download the software without registering an account (which compromises the implied anonymity of the review, or requires submitting fictitious personal data). Moreover, users with commercial email addresses cannot register - which is a minute detail, but still contravenes the Open Access/Open Source policy of the Journal. The software, even though it is (commendably) provided as a source code, comes with blanket reservation:*

*"Unless explicitly permitted by the RaptorX team, these standalone programs and datasets cannot be used for commercial purposes or included into a web server." in README file on the web server, where one can download the software. This alone makes the software not OSI-license compatible (*[*https://opensource.org/licenses/alphabetical*](https://opensource.org/licenses/alphabetical)*) which is a prerequisite for software papers in PLOS journals. The restriction of access, redistribution and usage violate the OSI Open Source Definition (*[*https://opensource.org/docs/osd*](https://opensource.org/docs/osd)*) at least in points 1, 3, 5, and 6.*

*Were authors to release the software with a free license, I would have no reservations.*

Answer: See our above answer.

*My remaining concerns are largely cosmetic:*

*p. 2 "protein data bank" -> "Protein Data Bank"*

Answer: thanks. We have fixed it.

*p. 4 "New Deep-learning Therader" -> "... Threader"*

Answer: we have fixed it.

*p. 9 The captions of Table 3 are still improperly typeset - which I trust will be fixed in the editorial office, but still makes the paper less approachable to read for reviewers.*

Answer: we have fixed it.

*p. 15 "Run TMalign to find ... " - it feels like there is a subject missing, consider adding "We".*

Answer: thanks for spotting this. We have fixed it.

*And general concern:*

*In many places authors contrast methods, asserting a supremacy of one over the other. Due to limited size of comparison sets, and often small margins of difference, it would greatly improve the paper, if there were confidence intervals or - even better - p-values provided with such comparisons.*

Answer: Thank you for your advice. We have calculated a p-value to measure the statistical significance of the difference between two competing methods.
